# Physiological and Molecular Responses to Excess Boron in *Citrus macrophylla* W

**DOI:** 10.1371/journal.pone.0134372

**Published:** 2015-07-30

**Authors:** Mary-Rus Martínez-Cuenca, Belén Martínez-Alcántara, Ana Quiñones, Marta Ruiz, Domingo J. Iglesias, Eduardo Primo-Millo, M. Ángeles Forner-Giner

**Affiliations:** 1 Department of Citriculture and Vegetal Production, Valencian Institute of Agrarian Research, Moncada, Valencia, Spain; 2 Department of Vegetal Protection and Biotechnology, Valencian Institute of Agrarian Research, Moncada, Valencia, Spain; University of Minho, PORTUGAL

## Abstract

This work provides insight into several mechanisms involved in boron (B) regulation pathway in response to high B conditions in *Citrus*. The study was carried out in *Citrus macrophylla* W. (Cm) seedlings cultured “in vitro” in media with 50 or 400 μM H_3_BO_3_ (control, Ct, and B-excess, +B, plants, respectively). Growth parameters, B concentration, leaf chlorophyll (Chl) concentration, the expression of the main putative genes involved in B transport and distribution, and leaf and root proline and malonaldehyde (MDA) concentrations, were assessed. Excess B led to high B concentration in +B plants (3.8- and 1.4-fold in leaves and roots, respectively) when compared with Ct ones. However, a minor effect was recorded in the plant (incipient visual symptoms, less than 27% reduction in root growth and 26% decrease in Chl *b* concentration). B toxicity down-regulated by half the expression level of putative B transporter genes *NIP5* and *PIP1*. *CmBOR1* gene was not repressed in +B plants and B accumulated in the shoots. High B level increased the transcripts of putative gene *TIP5*, involved in B transport across the tonoplast, by 3.3- and 2.4-fold in leaves and roots, respectively. The activity of V-PPiase proton pump, related with the electrochemical gradient in the vacuole, was also enhanced in +B organs. B toxicity up-regulated putative *BOR4* gene (2.1- and 2.7-fold in roots and leaves, respectively), which codifies for an active efflux B transporter. Accordingly, B was located in +B plants preferently in an insoluble form on cell walls. Finally, excess B caused a significant rise in proline concentration (51% and 34% in roots and leaves, respectively), while the MDA level did not exceed 20%. In conclusion, Cm tolerance to a high B level is likely based on the synergism of several specific mechanisms against B toxicity, including: 1/ down-regulation of NIP5 and PIP1 boron transporters; 2/ activation of B efflux from cells due to the up-regulation of putative *BOR4* gene; 3/ compartmentation of B in the vacuole through TIP5 transporter activation and the acidification of the organelle; 4/ insolubilisation of B and deposition in cell walls preventing from cytoplasm damage; and, 5/ induction of an efficient antioxidant system through proline accumulation.

## Introduction

Boron (B) is an essential micronutrient required in major physiological functions for the normal growth and development of higher plants [1]. This element participates in cell wall structure formation through the borate-diol bonding of two rhamnogalacturonan II molecules. It is also involved in root elongation, carbohydrate metabolism, phenol accumulation, pollen-tube growth and cell membrane integrity [2]. Boron excess occurs mostly by irrigation with high B level in water, or in arid and semiarid areas where water reaches topsoil by capillarity and then evaporates to cause B accumulation.

As boron accumulates in leaves as they age, B toxicity symptoms usually appear on older leaves, first as leaf tip and margin yellowing or mottling, then with a brownish burnt appearance, which finally ends in premature fall at high concentration levels [3]. It can also occasionally appear resinous gum spots on the undersides of leaves and it is as well associated with a shortened distance between leaf nodes. Therefore, severe B toxicity may induce loss of vigour, shorter branches, and even twig dieback.

It is widely accepted that *Citrus* is a sensitive crop to B excess, whose toxicity causes major disorders that lead to loss of yield [4]. However, further research is needed to better understand the regulation of the mechanisms involved in the whole response of citrus plants to the presence of high B levels in the media. Thus, after entering roots, boron is transported through xylem vessels, mostly bound to cell wall structures (insoluble B pool) or accumulated in apoplastic fluids (soluble B pool), while only another low soluble portion enters cells [4]. In plants, boron homeostasis seems to be related to the synergic regulation of several genes involved in B uptake, transport and partitioning in the aerial part [5]. Moreover, vacuolar compartmentation might also play a key role in B tolerance of cells [6].

On the molecular basis, the first B transporter identified in a biological system was *Arabidopsis thaliana* BOR1, an essential efflux-type B transporter for efficient loading of B in the xylem [7]. This gene is expressed mainly in root pericycle cells and its overexpression enhances root to shoot B translocation under B-limiting conditions [8]. When analysing the complete *A*. *thaliana* genome, six sequences were found to have a high homology with *AtBOR1* [1]. Among them, only *AtBOR4* has been characterised [9], and its overexpression markedly improves *A*. *thaliana* growth under high B conditions through B efflux. In fruit crops, *VvBOR1* and *CmBOR1* have been recently characterised as B transporters in *Vitis vinfera* and *Citrus macrophylla*, respectively [10,11].

Moreover, NIP5 is a boric acid channel that facilitates B influx to root cells in *A*. *thaliana* and *B*. *napus* [12,13]. It is localised to the plasma membrane on the outer side of epidermal, cortical and endodermal cells in roots. NIP5;1 is required for the uptake of B from the root surface and its accumulation is regulated in response to B deprivation [12]. In *A*. *thaliana*, the overexpression of *AtNIP5;1* results in root elongation under B-deficient conditions, which improves short-term B uptake [14]. Also, some evidences indicate that other PIPs aquaporins are also involved in B transport [15].

Finally, the vacuolar compartmentation of B has been related to the activity of *AtTIP5*, an aquaporin family member localised to the cell tonoplast membrane [16]. Under toxic B conditions, the *AtTIP5;1* overexpression has been reported to lower the cytoplasmic B concentration by accumulating B in the vacuole to, therefore, confer cell tolerance to toxic B levels [16]. Moreover, some proton pumps located in the vacuolar membrane, particularly tonoplast adenosine-triphosphatase subunit A (V-ATPase A) and tonoplast pyrophosphatase (V-PPiase), have been reported to be altered under different environmental stresses, such as B toxicity or salinity [17,18].

In addition to molecular regulation, some biochemical indicators related with oxidative stress and osmoprotective compounds, notably proline, have been well-defined in studies of plants subjected to different stresses [19,20]. The possible roles attributed to proline are balance of osmotic pressure, preservation of enzymes in the cytoplasm, detoxification of reactive oxygen species (ROS) and protection of membranes against lipid peroxidation [21]. However, data on proline and B toxicity are still scarce, and the role of this molecule regarding such nutritional stress is still not clear.

Citrus species differ in their susceptibility to B concentration [3,21]. *Citrus macrophylla* stands for a clementine species [22] and is classified as a potential tolerant one to B toxicity [23]. Hence the study of this plant’s global response when cultured under extreme B availability conditions could provide prospective insights into the mechanisms that determine *Citrus* tolerance to B excess. For this purpose, seedlings of *C*. *macrophylla* were cultured in media with normal B and at a high B level to study: A) the expression level of the main putative genes controlling B homeostasis within the plant under the above-mentioned conditions; B) boron distribution in different plant organs and compartmentation inside cells; C) the role of osmoprotective regulation against oxidative stress due to B excess.

## Materials and methods

### Ethics statement

The experiments were conducted in the Department of Citriculture and Vegetal Production from Valencian Institute of Agrarian Research (Moncada, Spain). Dr. Mary-Rus Martínez-Cuenca was response for experimental analysis in this manuscript and can be contacted in the future. The authors declare that this manuscript does not matter the any ethic issue and it does not involve endangered or protected species.

### Plant material and treatments

Seeds of *Citrus macrophylla* W. were sterilised for 5 min in a 2% v/v commercial bleach (0.5 M NaClO) solution prior to seed coat removal, rinsed 3 times with sterilized deionized water and transferred to a media containing distilled water (pH 6.0) with 0.4% agar added (Difco Bacto). Media were previously autoclaved at 120ºC for 20 min and distributed in 150 x 25-mm tubes (40 mL per tube). Seeds (one seed per tube) were germinated in a growth chamber (Sanyo MCR-350H, Sanyo Electric Biochemical Co, Japan) at 20-22/26-28ºC night/day temperatures, 80% relative humidity and 250 µmol m^-2^ s^-1^ photosynthetic photon flux density for 16 h per day.

After 20 days, seedlings with a single shoot were selected for uniformity and transferred individually after removing cotyledons in plastic 50 mL tubes containing a basic nutrient solution [1.5 mM Ca(NO_3_)_2_, 1.5 mM KNO_3_, 1 mM MgSO_4_, 1.2 mM H_3_PO_4_, 20 µM Fe-EDDHA, 7.65 µM ZnSO_4_·7H_2_O, 54.4 µM MnSO_4_·H_2_O, 0.55 µM MoO_3_, 0.5 µM CuSO_4_·5H_2_O] and 50 or 400 µM H_3_BO_3_ (Ct or +B, respectively). All the media were supplemented with 0.25% agar (pH 6.0) and sterilized as previously described. Seedlings were kept for 25 days under the same growth chamber conditions as described above.

### Plant growth

After 25 days of conditioning, seedlings were removed from culture tubes, rinsed with distilled water and divided into leaves, stems and roots. The length (in cm) of stems and roots was measured. Organs were fresh weighed and dried in a forced draft oven at 70ºC for 48 h until constant weight (DW, in mg).

### Total, soluble and insoluble B fractions

Roots and leaves samples were split into two subsamples each (0.5 g) and used for determination of B total (B_t_) and B fractions (B_f_) concentrations. Stem samples were used only for B_t_ determination. B_t_ concentration was measured according to [24]. Dry tissues (0.5 g) were burnt in a muffle furnace at 550ºC for 12 h. B was extracted with 0.1 N hydrochloridric acid, HCl, (Hiperpur Panreac) to a final volume of 5 mL. B_f_ (soluble in water, soluble in organic solvents and insoluble) concentration was measured according to [25]. Samples were frozen and ground into fine powder in liquid nitrogen (N_2_) by a mortar. The powder was homogenized with 10 volumes of ice-cold water and centrifuged at 1,000 rpm for 10 min. The precipitate was washed with 10 volumes of ice-cold water and re-centrifuged. The combined supernatants were defined as water-soluble B fraction, which is B mainly found in the cells free space. The residue was washed 3 times with 10 volumes of 80% ethanol, once with 10 volumes of methanol:chloroform mixture (1:1, v/v) and once with 10 volumes of acetone, to extract B inside the protoplast and apparently linked to organic molecules. The insoluble pellet was used for B bound to cell wall. All fractions were dried and ashed similarly as B_t_ samples. B_t_ and B_f_ concentrations were determined by inductively coupled plasma atomic emission spectroscopy (ICP-AES iCAP 6000, Thermo Scientific).

### Leaf Chl concentration

Leaf chlorophyll (Chl) concentration was measured according to Moran and Porath [26]. Samples from the two youngest fully expanded leaves per plant were separately ground with a mortar in N_2_. Fresh material (0.05 g) was incubated in 4 mL N,N-dimethylformamide at 4ºC for 72 h and centrifuged at 4,000 rpm and 4ºC for 15 min (Eppendorf Centrifuge 5810R, AG, Hamburg, Germany). Supernatant was left for 1 h in the presence of Na_2_SO_4_ and the absorbance was measured at 664 and 647 nm (Lambda 25, PerkinElmer, Shelton, CT, USA). The average value of the two leaves was considered representative of each plant.

### Proline and MDA concentration

Free-proline concentration in leaves and roots was determined according to Delauney and Verma [19]. Fresh tissue was ground with a mortar in N_2_. Sample material (0.1 g) was homogenized (Vortex) in 1.5 mL sulphosalicylic acid (3%) for 1 min, centrifuged at 14,000 rpm for 5 min (Eppendorf Centrifuge 5810R, AG, Hamburg, Germany) and the supernatant was stored at 4ºC. An aliquot (0.2 mL) was incubated with 0.5 mL sulphosalicylic acid (3%), 0.7 mL reactive ninhydrin acid reagent (ninhydrin, phosphoric acid 6 M, glacial acetic acid 60%) and 0.6 mL glacial acetic acid (99%) in a dry bath at 100ºC for 1 h (Thermostatic Bath BD, Bunsen SA, Humanes, Spain). Samples were cooled down in an ice bath for 15 min and absorbance was measured at 520 nm (SmartSpec Plus, Bio-Rad, West Berkeley, California, USA).

Lipid peroxidation in leaves and roots was determined by measuring the malondialdehyde (MDA) concentration according to Hodges et al. [27]. Frozen sample material (0.1 g) was homogenized (Polytron PT 3100, Lucerne, Switzerland) in 5 mL of cold ethanol (80%) at 10,000 rpm for 1 min, centrifuged at 3,000 rpm for 30 min and 4ºC (Eppendorf Centrifuge 5810R, AG, Hamburg, Germany) and the supernatant was stored at 4ºC: (a) an aliquot (1 mL) was combined with an equal volume of a 20% trichloroacetic solution (TCA), while (b) another aliquot (1 mL) was combined with an equal volume of a 0.5% thiobarbituric acid (TBA) and 20% TCA solution. Test tubes were covered with glass marbles to avoid evaporation and incubated at 90ºC for 1 h in a water bath (Thermostatic Bath BD, Bunsen SA, Humanes, Spain). Samples were cooled down in an ice bath for 15 min and centrifuged at 1,000 rpm for 15 min and 4ºC (Eppendorf Centrifuge 5810R, AG, Hamburg, Germany). Absorbance of supernatant (a) was measured at 532 and 600 nm (SmartSpec Plus, Bio-Rad, West Berkeley, California, USA) using 20% TCA as a blank. Absorbance of supernatant (b) was measured at 440, 532 and 600 nm using a solution of 0.5% TBA and 20% TCA as a blank. Concentration of MDA was calculated using an extinction coefficient of 155 mM^-1^ cm^-1^.

### RNA extraction and real-time RT-PCR analysis

Total plant RNA from leaf and root tissues was extracted using the RNeasy Plant Mini Kit (Qiagen, Hilden, Germany). Fresh samples (0.1 g) were ground with a mortar in N_2_. To remove genomic DNA, RNA samples were treated with RNase free DNase (Qiagen) following the manufacturer’s instructions. RNA quality and concentration were assessed with ND-1000 full spectrum UV-Vis spectrophotometer (Nanodrop Technologies, Thermo Fisher Scientific, Delaware, USA). Quantitative real-time polymerase chain reactions (RT-PCR) were run in a LightCycler 2.0 Instrument (Roche, Diagnostics GmbH, Mannheim, Germany) equipped with version 4.0 of the Light Cycler Software. Reactions contained 2.5 units of MultiScribe Reverse Transcriptase (Applied Biosystems, Roche Molecular Systems, New Yersey, USA), 1 unit of RNase Inhibitor (Applied Biosystems), 2 µL of LC Fast Start DNA Master PLUS SYBR Green I (Roche), 25 ng of total RNA, and 250 nM of the specific forward and reverse primers in a total volume of 10 µL. The PCR programme was run at 48ºC for 30 min, 95ºC for 10 min, followed by 45 cycles at 95ºC for 2 s, 58ºC for 8 s and 72ºC for 8 s. The fluorescent intensity data were acquired during the 72ºC extension step and were transformed into relative mRNA values using a 10-fold dilution series of an RNA sample as a standard curve. The relative mRNA levels were normalized to total RNA amounts as previously described [28]. Actin was used as the reference gene [29] and the specificity of the amplification reactions was assessed by post-amplification dissociation curves and by sequencing the reaction product. At least three independent RNA extractions and real-time reactions with three technical replicates per sample were performed. Putative *BOR4 and TIP5* genes were identified by a homology search in the clementine genome full-length Phytozome v9.0 database [30]. The expression levels of *NIP5*, *PIP1*, *PIP2*, *CmBOR1*, *V-PPiase* and *V-ATPase A* genes were evaluated using the forward and reverse primers described by other authors [11,31,32,33]. Details of the forward and reverse primers used in the RT-PCR are listed in Table 1. Gene-predicted products ranged from 75 to 156 bp.

**Table 1:** List of the primers used for quantitative real-time PCR.

Annotation	Code	Forward/reverse primer (5'-3')	Reference
*NIP5*	JQ277272 ^1^	AGCTTCCCGGATATTCCAGT	[31]
		ACTATGGGTCCTGCTGTTGC	
*CmBOR1*	EF581174 ^1^	GGGCATATAGTCCCCGTGTT	[11]
		CCGGGACTGGGAACTTTC	
*PIP1*	Ciclev10012384^2^	AGGATTACACGGAGCCACCT	[33]
		TGCTTTTGGATTTGGACACG	
*PIP2*	Ciclev10029003 ^2^	TGTGTTCATGGTTCACTTGG	[33]
		TGAATGGTCCAACCCAGAAG	
*BOR4*	Ciclev10019073^2^	CTGAAAAGCCGAAAAGCAAG	
		CACGCCGTAGTCTGCTATGA	
*TIP5*	Ciclev10006865^2^	CCGGAAGTTTCAAAAACCAG	
		ACCGCAGTTCCATCAGAAAC	
*V-PPiase*	JN580556^1^	GCATACAGCCCTGTGCAAGATA	[32]
		CCTCCAGCATTGTCACTGATG	
*V-ATPase A*	JN580555^1^	TCCTGTAACATCTGCCACCCTCA	[32]
		TTATCTCCTTCAGCCAAAGCATC	

^1^Accession number in NCBI reference sequence database (http://www.ncbi.nlm.nih.gov).

^2^Code refers to the transcript name in the database available in the International Citrus Genome Consortium (http://www.phytozome.net/search.php).

### Statistical analyses

For statistical analyses, the values of growth parameters, chlorophyll concentration, proline and MDA concentrations are the mean of six independent plants per treatment. Values from other analyses are the mean and the standard deviation of at least three replicates from three independent groups of ten plants per treatment. Data were submitted to an analysis of variance (ANOVA) with Statgraphics Plus, version 5.1 (Statistical Graphics, Englewood Cliffs, NJ), prior to testing for normality and homogeneity. When the ANOVA showed a statistical effect, means were separated by least significant differences (LSD) at *P <* 0.05.

## Results and Discussion

### Symptoms, plant growth parameters, total B and chlorophyll concentrations

**Fig 1:**
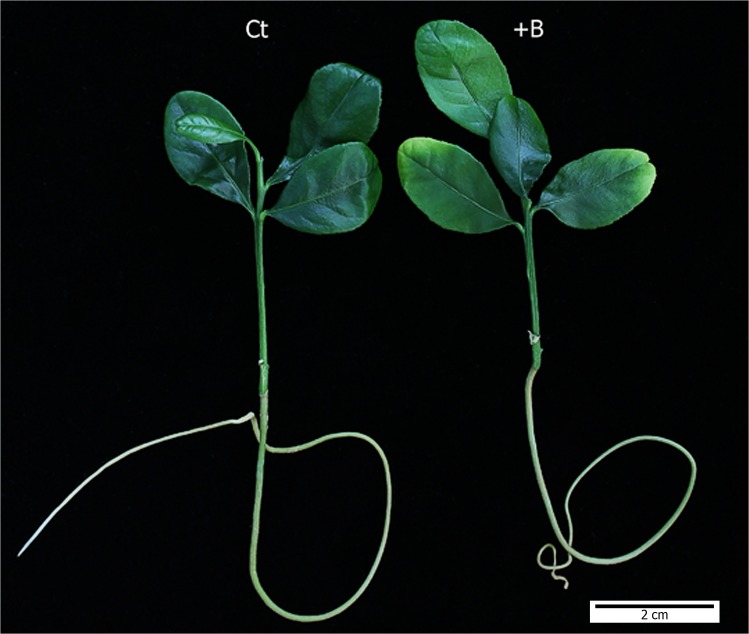
Visual symptoms in leaves of *Citrus macrophylla* seedlings grown for 25 days in B-normal (50 μM, Ct) and B-toxic (400 μM, +B) nutrient solutions.

Twenty-five days after plants were grown in the presence of 400 μM H_3_BO_3_, +B plants showed incipient symptoms of B toxicity on a few leaves (Fig 1), consisting in small yellowish zones near their apical end and borders resembling those previously reported for other citrus species [3,21].

Regarding plant growth, +B plants showed significant reductions in root dry weight and root length of 27.3% and 14.0%, respectively, related to Ct ones (Table 2), while no significant differences in growth parameters were observed in the shoots. Stunted root growth is a typical symptom in plants grown under B-excess conditions [21,34]. However, this effect was not evidenced in the shoot, which seems capable of maintaining a relatively high growth index despite accumulating large B concentrations in leaves, as occurs in some *Solanum* and *Puccinellia* plants considered to be B toxicity tolerants [35,36].

**Table 2:** Growth parameters [dry weight (DW, mg) and length (cm)], total boron concentration ([B_t_], μg g^-1^ DW) and total B content (B_t_, μg) measured in roots, stems and leaves of *Citrus macrophylla* seedlings grown for 25 days in B-normal (50 μM, Ct) and B-toxic (400 μM, +B) nutrient solutions.

	Root				Stem				Leaf			
	Ct		+B		Ct		+B		Ct		+B	
DW (mg) ^1^	12.8±1.1	a	9.3±0.2	b	2.9±0.1		2.7±0.2		13.3±0.9		12.8±0.5	
Length (cm) ^1^	15.7±1.6	a	13.5±0.7	b	4.0±0.3		3.5±0.3					
[B_t_] concentration(μg g^-1^ DW) ^2^	39.4±3.1	b	55.6±5.4	a	34.5±2.8	b	57.9±5.7	a	92.2±7.4	b	345.8±22.4	a
B_t_ content (μg) ^2^	504±70		517±45		98.±9	b	153±13	a	1226±101	b	4426±326	a

^1^Values of DW and length are the means ± SE of six independent plants per treatment (n = 6).

^2^Values of [B_t_] and B_t_ are the means ± SE of three independent experiments (n = 3).

For a comparison of means, an ANOVA followed by the LSD test, calculated at the 95% confidence level, was performed. Different letters indicate significant differences for each parameter and within each plant organ (*P <*0.05).

Moreover, +B plants registered a substantial rise in B concentration in the leaves (3.8-fold) when compared with Ct ones (Table 2) and reached a level close to 350 μg g^-1^ DW, which is considered to fall within the excess range (> 250 μg g^-1^ DW; [37]). Roots from +B seedlings also showed higher B concentration (1.4-fold increase) than Ct ones, being much lower than that observed in leaves (Table 2).

**Fig 2:**
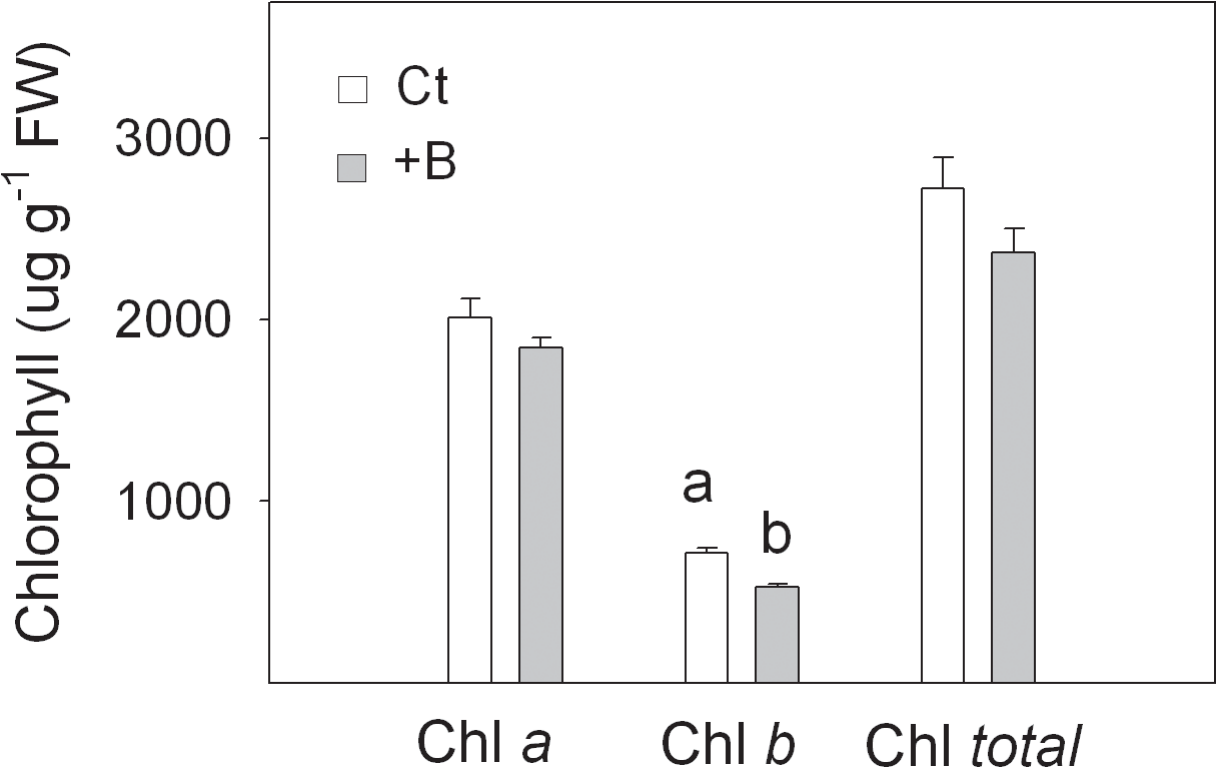
Chlorophyll (Ch) *a*, *b* and *total* content (µg g FW^−1^) measured in fully developed leaves of *Citrus macrophylla* seedlings grown for 25 days in B-normal (50 μM, Ct) and B-toxic (400 μM, +B) nutrient solutions. Values are the means ± SE of six independent plants per treatment (n = 6). For a comparison of means, an ANOVA followed by the LSD test, calculated at 95% confidence level, was performed. Different letters in the same row indicate significant differences between treatments (*P <*0.05).

Finally, we also determined chlorophylls concentration (*a*, *b* and *total*) in leaves as stress indicator to check the degree of tolerance of Cm seedlings to B toxicity in citrus [3,38]. The results indicated that B excess only led to a significant 26.3% reduction of Chl *b* values in +B seedlings when compared to Ct ones (Fig 2).

The data of shoot growth and chlorophyll concentration in leaves, together with the incipient B toxicity symptoms shown by the seedlings cultured at a high external B level, suggest that, despite the elevated B concentration reached by leaves, Cm behaves as a B-excess-tolerant plant. Therefore, the present work studied some of the mechanisms that likely enable Cm seedlings to tolerate B excess in accordance with previous proposals [11,21].

### Transcription analysis of candidate genes related with B homeostasis in the plant

#### Expression of putative *NIP5*, *PIP1* and *PIP2* genes and its relation with B transport in the plant

**Fig 3:**
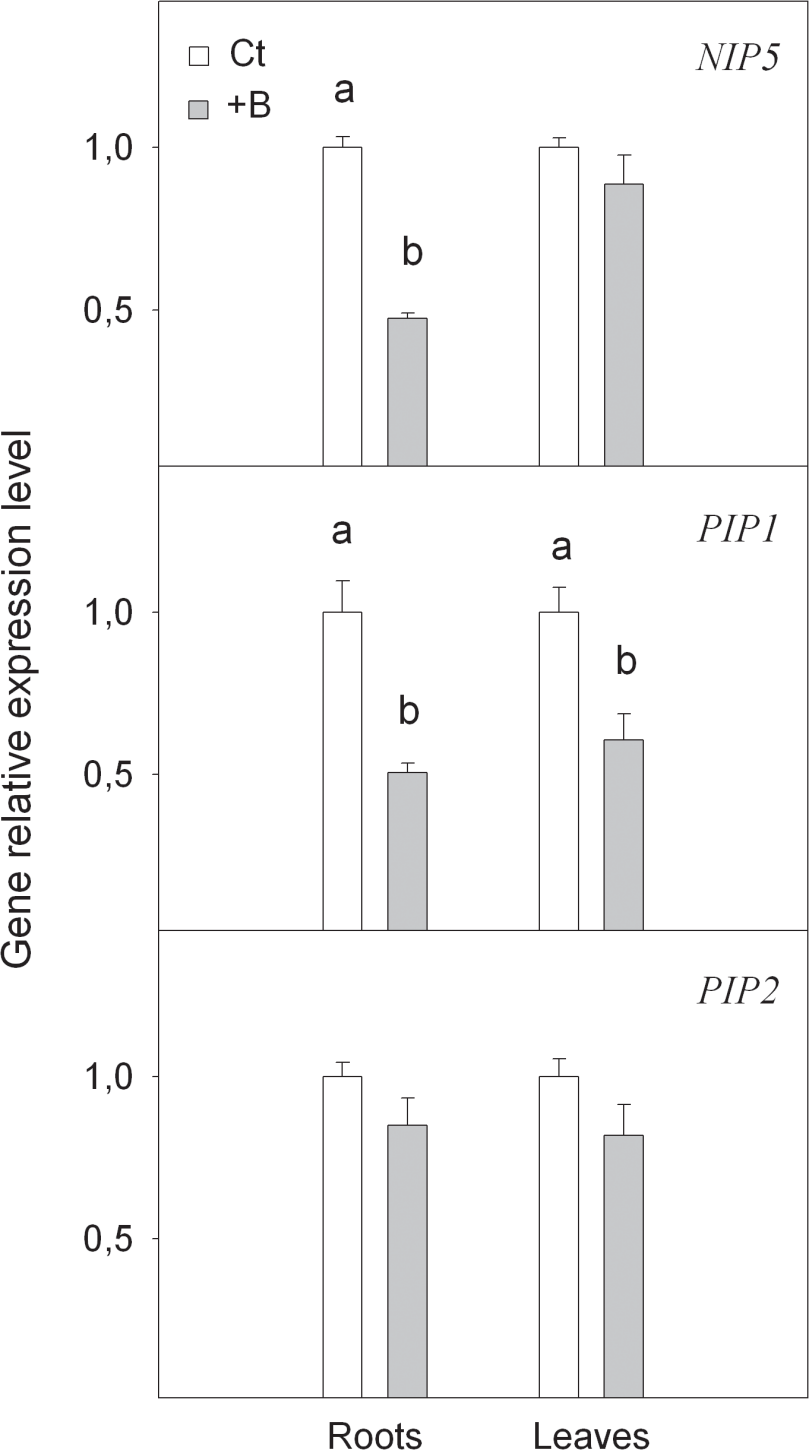
Relative expression of *NIP5*, *PIP1* and *PIP2* genes measured by real-time RT-PCR analysis, in roots and leaves of *Citrus macrophylla* seedlings grown for 25 days in B-normal (50 μM, Ct) and B-toxic (400 μM, +B) nutrient solutions. Values are the means ± SE of three independent experiments (n = 3). For a comparison of means, an ANOVA followed by the LSD test, calculated at 95% confidence level, was performed. Different letters in the same organ indicate significant differences between treatments (*P <*0.05).

Acquisition of boron by cells is associated with the activity of some members of the aquaporin family codified by the genes *NIP5*, *PIP1* and *PIP2* (Fig 3). The expression level of putative *NIP5* gene lowered in roots as a result of high B levels. Thus, +B seedlings contained 52% fewer *NIP5* transcripts in roots than Ct ones. It is noteworthy that no effect on the *NIP5* transcript level was recorded when analysed in +B leaves. In *A*. *thaliana*, this gene codified a boric acid channel for efficient B acquisition under limited B supply [12]. This result was also found in Carrizo citrange roots, where *CiNIP5* expression increased under B-deficiency conditions [31]. In contrast, its down-expression under B-excess conditions in the roots of *Citrus* plants has revealed a point of regulation of B uptake efficiency of different citrus rootstocks. Similar behaviour had been previously described by Schnurbusch et al. [39], which showed a relation between *HvNIP2*:*1* gene expression and B toxicity tolerance in barley. Moreover, Tanaka et al. [40] suggested the importance of *AtNIP5*:*1* degradation for plant acclimation to high B conditions.

*PIP1* expression level was also reduced when analyzed in +B seedlings related to Ct ones (Fig 3). In particular, +B plants registered a 50% and 40% reduction in *PIP1* transcript levels, in leaves and roots respectively, when compared with Ct ones. This indicates the possibility of reduced cell permeability to B under excess conditions. In this way, the expression of *ZmPIP1* in *Xenopus laevis* oocytes indicates that this aquaporin is involved in B transport within cells [41]. In contrast, no differences in the *PIP2* expression level were found between Ct and +B treated seedlings (Fig 3).

#### Expression of *CmBOR1* gene under B toxicity conditions and its relation with B transport in the plant

**Fig 4:**
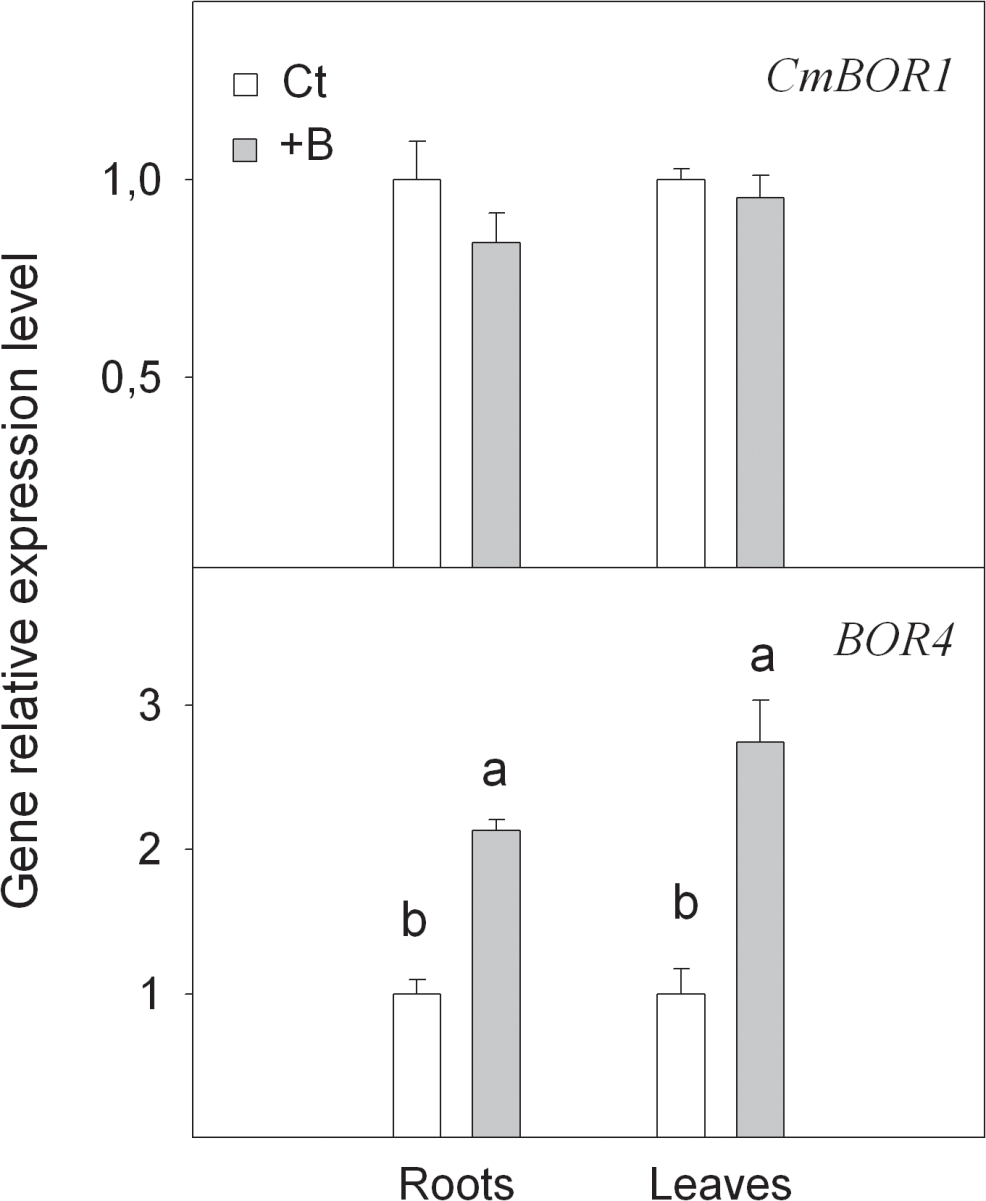
Relative expression of *CmBOR1* and *BOR4* genes measured by real-time RT-PCR analysis, in roots and leaves of *Citrus macrophylla* seedlings grown for 25 days in B-normal (50 μM, Ct) and B-toxic (400 μM, +B) nutrient solutions. Values are the means ± SE of three independent experiments (n = 3). For a comparison of means, an ANOVA followed by the LSD test, calculated at 95% confidence level, was performed. Different letters in the same organ indicate significant differences between treatments (*P <*0.05).

We also monitored the expression level of *CmBOR1* gene (Fig 4), which has been reported to modulate B transport in plants due to its role in loading B into the xylem, and therefore to translocate B from roots to shoots [7,12]. However, no differences in the *CmBOR1* expression level in the leaves and roots of Ct and +B seedlings were found. This is in accordance with the high capacity of *Citrus* to accumulate B in their leaves at high external B levels, which suggests that citrus plants have an effective system for B transport from roots to leaves, even under B-excess conditions. Therefore, the presented data indicate that B toxicity tolerance is not linked to a repressible B transport mechanism. To support this, it has been reported that the *CmBOR1* transcript level is not affected by high B supply and the activity of this gene does not prevent high B accumulation in citrus leaves [11]. In *Oryza sativa*, OsBOR1 loads B into the xylem and also participates in the absorption of this element in roots [42]. In *Brassica napus*, *BnBOR1;1c* and *BnBOR1;2a* are up-regulated in roots under low B stress, but no differences in their expression have been found between B-efficient and B-inefficient cultivars in low or normal B environments [13]. However, some evidence has indicated that the removal of the BOR1 protein from membranes and its rapid degradation through the endocytic pathway may occur in response to high B levels in *A*. *thaliana* [43]. This suggests that this mechanism in other species could be involved in the regulation of B transport from roots to shoots at high B levels.

#### Accumulation of putative *BOR4* transcripts under B toxicity conditions

The transcript level of the putative *BOR4* gene was considerably enhanced in the roots and leaves of +B seedlings (2.1- and 2.7-fold increase, respectively) when compared with Ct ones (Fig 4). *BOR4* gene has been reported to codify for a transporter to allow an active efflux of B across the plasma membrane [44]. *BOR4* gene overexpression might lead to B excess to be pumped out of the cell and mitigate B toxicity stress in the cytoplasm. It is likely that a large amount of B exported from cells remains linked to the cell wall. In *Arabidopsis*, GFP fluorescence derived from BOR4-GFP strongly localised BOR4 to the plasma membranes of the distal sides of epidermal cells in the elongation zone. This is of strategic importance for directional export of B to avoid high concentrations in the xylem and growing cells [9] and likely to limit B accumulation in the symplasm. *BOR4* is likely to operate at high B concentrations (low-affinity transporter), but not to mediate B efflux under low B conditions. To support this notion, it has been reported that high B-tolerant barley and wheat cultivars are able to maintain low B concentrations in shoots and roots by active B efflux mediated by the overexpressions of *Hv-BOR2* and *Ta-BOR2* [45], which suggests a positive correlation between these genes and the degree of tolerance among different cultivars in these species.

#### Accumulation of putative *TIP5* transcripts under B toxicity conditions and its relation with B compartmentation in the vacuole

The expression of the *TIP5* gene coding for a B transporter related with B sequestration across the tonoplast, and *V-ATPase A* and *V-PPiase* genes coding for two H^+^-ATPases pumps in the vacuole membrane, were checked to know if increased B accumulation in the vacuole could constitute a mechanism of B toxicity tolerance.

**Fig 5:**
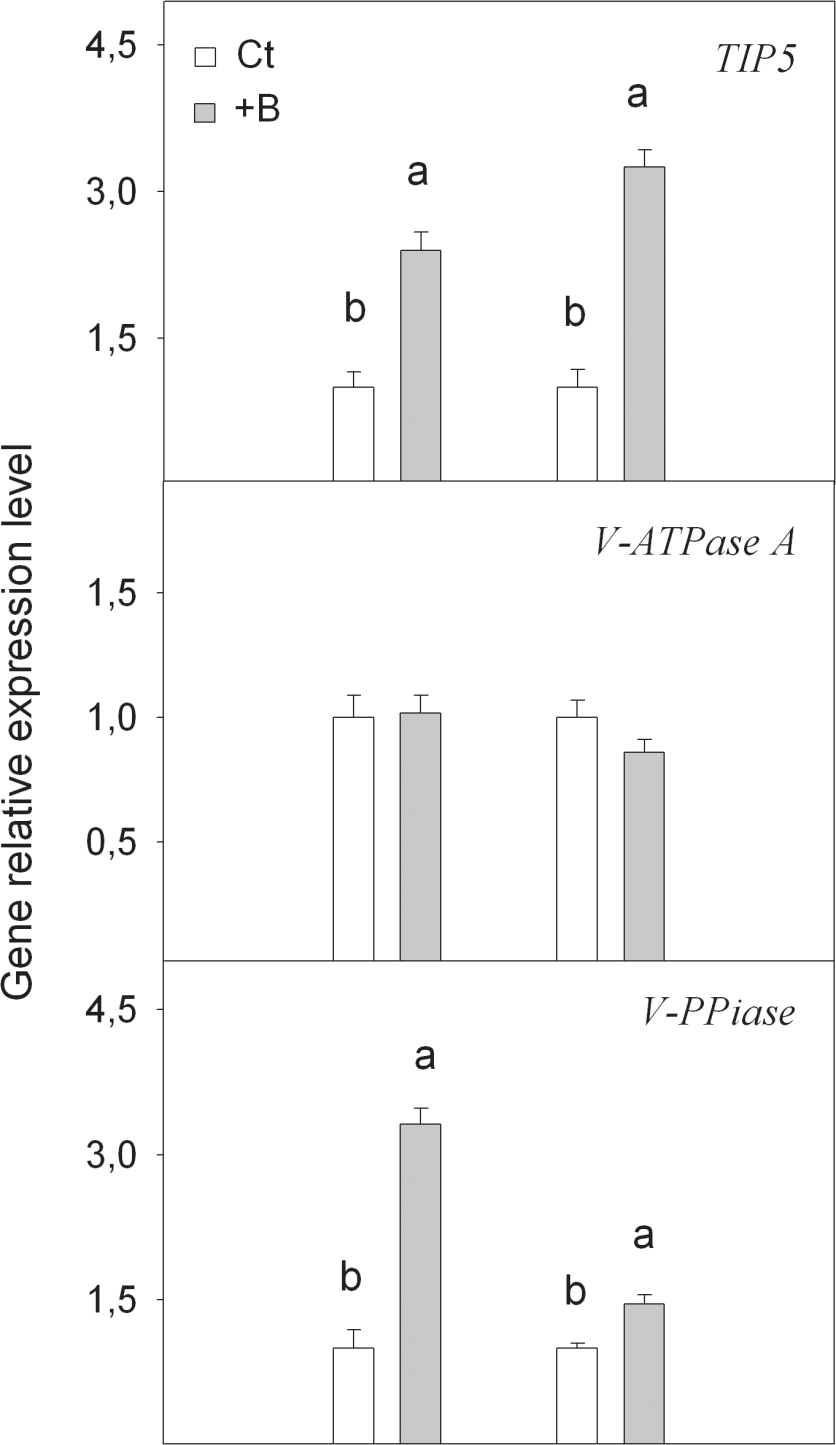
Relative expression of *TIP5*, *V-PPiase* and *V-ATPase A* genes measured by real-time RT-PCR analysis, in roots and leaves of *Citrus macrophylla* seedlings grown for 25 days in B-normal (50 μM, Ct) and B-toxic (400 μM, +B) nutrient solutions. Values are the means ± SE of three independent experiments (n = 3). For a comparison of means, an ANOVA followed by the LSD test, calculated at 95% confidence level, was performed. Different letters in the same organ indicate significant differences between treatments (*P <*0.05).

In this study, boron excess promoted the expression level of putative gene *TIP5*, and the number of transcripts increased in the leaves and roots of +B seedlings by 3.3- and 2.4-fold, respectively, in comparison with Ct ones (Fig 5). Aquaporin TIP5 has been related to a decreasing B concentration in the cytoplasm through its transport across the tonoplast membrane and to compartmenting it into the vacuole complexed with B-polyols [6]. Thus at high B levels in culture media, the up-regulation of putative gene *TIP5* might be responsible for the intracellular B allocation, which could contribute to B tolerance in *Citrus*. Pang et al. [16] pointed out this capacity in *A*. *thaliana*, and provided evidence to support that this gene may be involved in B transport to vacuoles. Vacuolar sequestration has also been widely assumed to play a fundamental role in plant tolerance to excess micronutrients and heavy metals [46].

A raised expression level of *V-PPiase* in +B organs, in comparison to those of Ct plants, suggests that V-PPiase is likely involved in the acidification of the vacuole while no effect was recorded on V-ATPase A. The protonmotive force generated by this vacuolar H^+^-translocating enzyme, enhances antiporters involved in B transport to increase their activity and, consequently, B sequestration into this organelle is more efficient, similarly to what is described for sodium tonoplast antiporters during salt stress conditions [47,48]. Maybe even the action of putative tonoplast antiporters of polyols (due to their ability to complex with B) might play here as secondary transporters energized by the tonoplast H^+^ gradient and its role should not be discarded.

The synergism of both responses in the vacuole will probably help to the active B influx in this organelle through the tonoplast, alleviating from B toxic concentrations in the cytoplasm.

### Cellular B allocation under B toxicity conditions: partitioning in soluble and insoluble B fractions in roots and leaves

**Fig 6:**
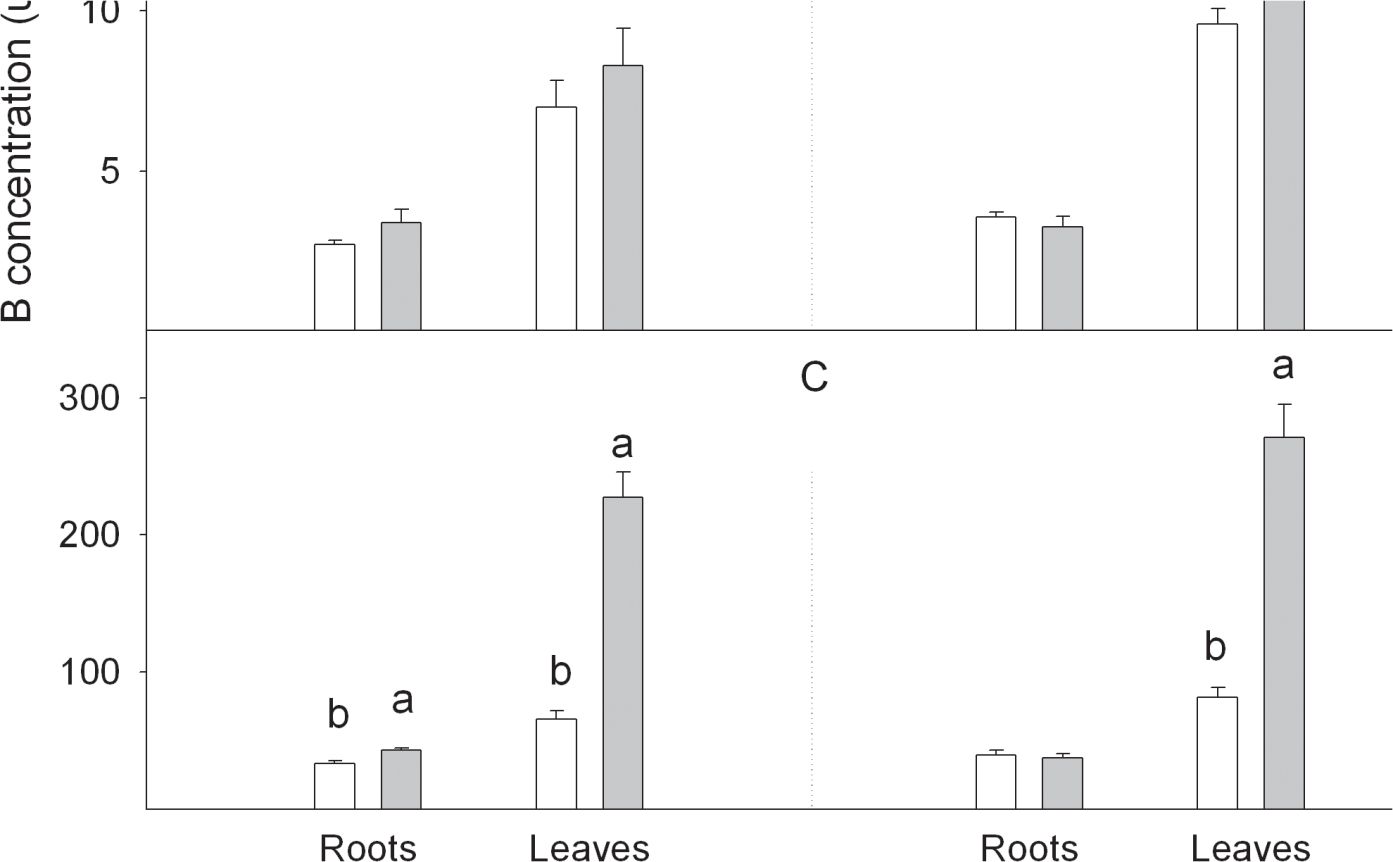
Boron concentration ([B_f_], μg g^-1^ DW) and boron content (B_f_, μg) in (A) soluble in water, (B) soluble in organic solvents and (C) insoluble fractions measured in roots and leaves of *Citrus macrophylla* seedlings grown for 25 days in B-normal (50 μM, Ct) and B-toxic (400 μM, +B) nutrient solutions. Values are the means ± SE of three independent experiments (n = 3). For a comparison of means, an ANOVA followed by the LSD test, calculated at the 95% confidence level, was performed. Different letters indicate significant differences for each parameter and within each plant organ (*P <*0.05).

Plants contain boron in both soluble and insoluble forms [4]. According to Liu et al. [4], B extracted in water is likely to be localized in the plant free space, or apoplast, while the remaining soluble B, extracted using organic solvents, belongs to B located inside cells or the protoplast and linked to sugars, alcohols and polyhydroxycarbolates [49]. Both fractions represent not only mobile B, but also the only form in plant tissues that can be re-translocated in the phloem [5]. Finally, the B-insoluble fraction represents the B bound to cell walls linked to peptic polysaccharides [25]. Fig 6 shows the partitioning of B in soluble (water and organic solvents) and insoluble forms in roots and leaves. Insoluble B (Fig 6C) was mostly the main fraction of B in both Ct and +B seedlings (more than 65.8% of total B), followed by the water-soluble fraction (ranging from 31.8% to 9% of total B; Fig 6A), and lastly by soluble B in the organic solvents fraction (below 7.6%; Fig 6B). However, the seedlings grown under B-excess conditions increased the concentration and total amount of B located in cell walls and in the soluble fraction from the apoplast when compared with Ct plants, which is likely in equilibrium with the external medium. Nevertheless, the concentration and the amount of B extracted with organic solvents, which is attributed to cytoplasmic B, was similar in the organs from both +B and Ct seedlings. As boron exerts its toxicity inside rather than outside cells, the capability to retain increased B amounts linked to cell walls under B-excess conditions enables this genotype to block excess B in an insoluble form, thus preventing its entry to the cytoplasm to protect cells from B toxicity. Accordingly, Nozawa et al. [50] found a negative correlation between the B concentration inside yeast cells and the degree of tolerance to a high external B level, and concluded that cells with lower protoplasmic B were more tolerant to high B levels. Other authors have also observed a lower B concentration in leaf protoplasts of B-toxicity-tolerant cultivars of barley and wheat, which suggests different B partitioning within the shoot as a tolerance mechanism [42]. Hence it is likely that insolubilisation of B outside the cytoplasm might constitute another mechanism of B toxicity tolerance.

### Accumulation of proline in roots and leaves: possible role in antioxidant response to MDA concentration

Boron effects on proline concentration have been studied in organs since this amino acid usually accumulates in response to several abiotic stresses [19,20]. Boron excess caused a significant rise in the proline concentration recorded in the roots and leaves of +B seedlings (50.7% and 33.6%, respectively) when compared to Ct ones (Table 3). Among other functions, proline acts as a detoxifier of hydroxyl radicals in plants [19,51]. Accordingly, proline levels enhanced by high B concentrations have been recorded in tomato and pepper [35,52]. MDA concentration increased by approximately 18.3% and 20.2% in the roots and leaves, respectively, of +B seedlings when compared with Ct ones (Table 3). The reduced effect of excess B on MDA concentration detected in both organs of +B seedlings indicates a low response to oxidative stress under B toxicity. Likewise, Gimeno et al. [21] did not find any effect on MDA concentration measured in the roots and leaves of Verna lemon trees when grown at high B levels.

**Table 3:** Proline (µmol g FW^−1^) and malonaldehyde (MDA, nmol g FW^−1^) concentrations measured in roots and leaves of *Citrus macrophylla* seedlings grown for 25 days in B-normal (50 μM, Ct) and B-toxic (400 μM, +B) nutrient solutions.

	Ct		+B	
Proline (µmol g FW^−1^)^1^				
Root	487±28	b	734±44	a
Leaf	660±56	b	882±45	a
MDA (nmol g FW^−1^)^1^				
Root	251±24	b	297±12	a
Leaf	426±36	b	512±36	a

^1^Values are the means ± SE of six independent plants per treatment (n = 6).

For a comparison of means, an ANOVA followed by the LSD test, calculated at 95% confidence level, was performed. Different letters in the same row indicate significant differences between treatments (*P <*0.05).

It has been reported that proline levels are inversely related with MDA concentration in the leaves of several woody species grown under B-excess conditions [38,53,54]. Accordingly, it was described that elevated proline avoids the generation of free radical levels as measured by MDA [55,56]. This coincides with the data presented herein and suggests that, at high external B concentration, Cm seedlings display a sharp rise in proline levels in both leaves and roots, whereas the increased MDA concentration was much lower. This suggests the existence of an efficient antioxidant system in Cm that is able to cope with ROS species generated as a result of B toxicity. As reported by Xiong and Zhu [56], proline is capable of detoxifying ROS by forming a stable complex with them to, thus, inhibit the lipid peroxidation process. This points at the role of this amino acid as part of a likely efficient antioxidant system able to cope with ROS species generated as a result of B toxicity in Cm plants. However, the function of other key antioxidant enzymes as catalase, superoxide dismutase and glutathione reductase should not be discarded [34,57], as they might also play an important role in the efficiency of the whole antioxidant system of the plant.

## Conclusion

In *Citrus*, boron exerts its toxicity more markedly in leaves than in root system. However, the low degree of B toxicity symptoms in Cm leaves indicates that this species substantially behaves as a tolerant plant to high B levels. This aptitude is likely based on: A) the down-regulation of the main B transport channels in the root, *NIP5* and *PIP1* genes, which blocks the plant’s B uptake capacity; B) although B transport faculty is not repressed, the overexpression of putative gene *BOR4*, facilitates the export of B excess from cells and avoids cytoplasm damage; C) the capacity to hold B in an insoluble form in the leaves mainly allocated in cell walls; D) the compartmentalization of toxic B from the cytoplasm inside the vacuole due to the up-regulation of aquaporin *TIP5* and the induction of the protonmotive gradient in the tonoplast; and, E) the activation of an antioxidant system against oxidative stress through proline accumulation.
