# Correction: Physiological and Molecular Responses to Excess Boron in Citrus macrophylla W

**DOI:** 10.1371/journal.pone.0137941

**Published:** 2015-09-04

**Authors:** Mary-Rus Martínez-Cuenca, Belén Martínez-Alcántara, Ana Quiñones, Marta Ruiz, Domingo J. Iglesias, Eduardo Primo-Millo, M. Ángeles Forner-Giner

Fig 6, “Boron concentration ([B_f_], μg g^-1^ DW) and boron content (B_f_, μg) in (A) soluble in water, (B) soluble in organic solvents and (C) insoluble fractions measured in roots and leaves of *Citrus macrophylla* seedlings grown for 25 days in B-normal (50 μM, Ct) and B-toxic (400 μM, +B) nutrient solutions,” appears incorrectly. Please see the corrected Fig 6 here.

**Fig 6:**
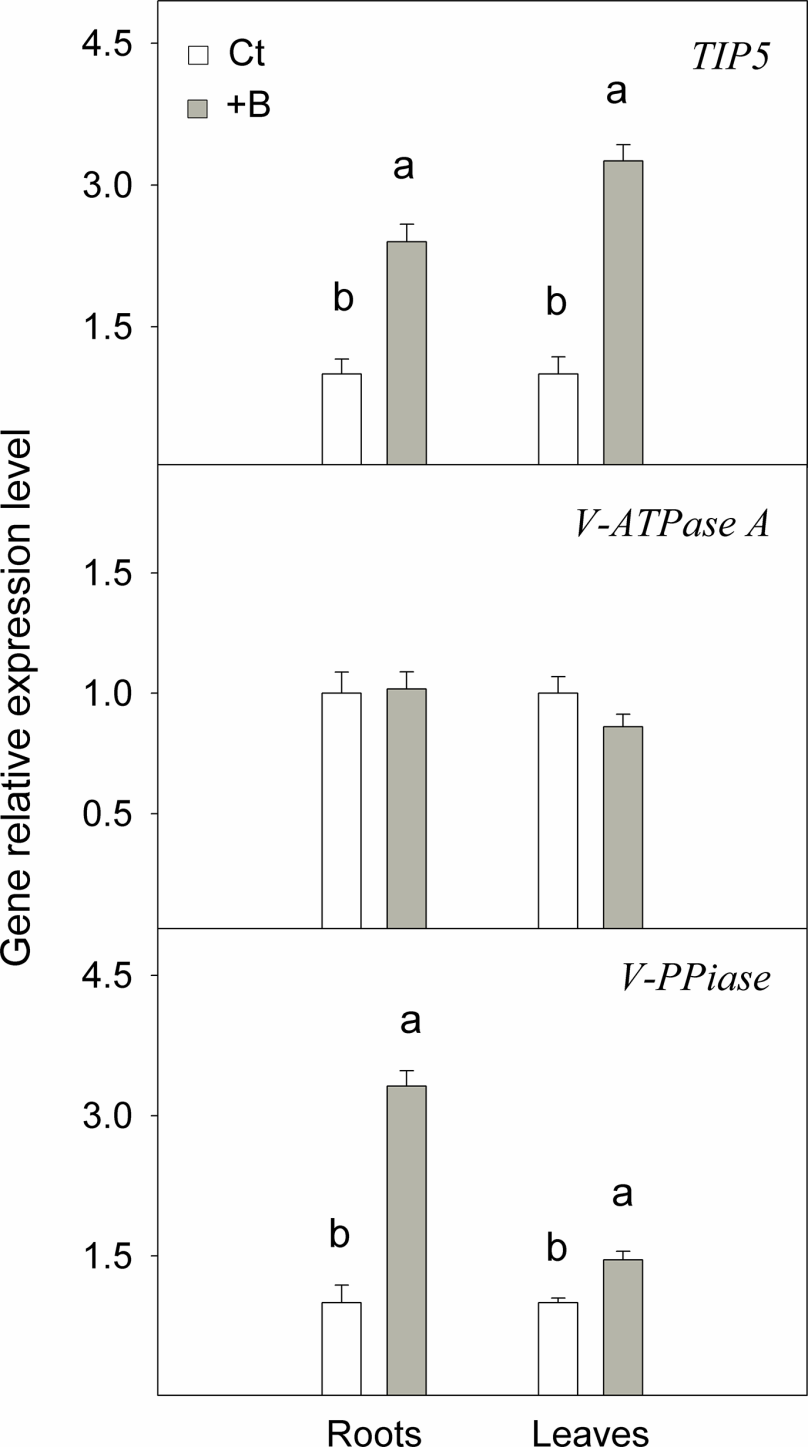
Boron concentration ([B_f_], μg g^-1^ DW) and boron content (B_f_, μg) in (A) soluble in water, (B) soluble in organic solvents and (C) insoluble fractions measured in roots and leaves of *Citrus macrophylla* seedlings grown for 25 days in B-normal (50 μM, Ct) and B-toxic (400 μM, +B) nutrient solutions. Values are the means ± SE of three independent experiments (n = 3). For a comparison of means, an ANOVA followed by the LSD test, calculated at the 95% confidence level, was performed. Different letters indicate significant differences for each parameter and within each plant organ (P <0.05).
