# Correction: Correction: Physiological and Molecular Responses to Excess Boron in *Citrus macrophylla* W

**DOI:** 10.1371/journal.pone.0142358

**Published:** 2015-11-03

**Authors:** The PLOS ONE Staff

The incorrect image for Fig 6 was included in the correction published on September 4, 2015. The publisher apologizes for the error. Please view the correct Fig 6 here.

**Fig 6:**
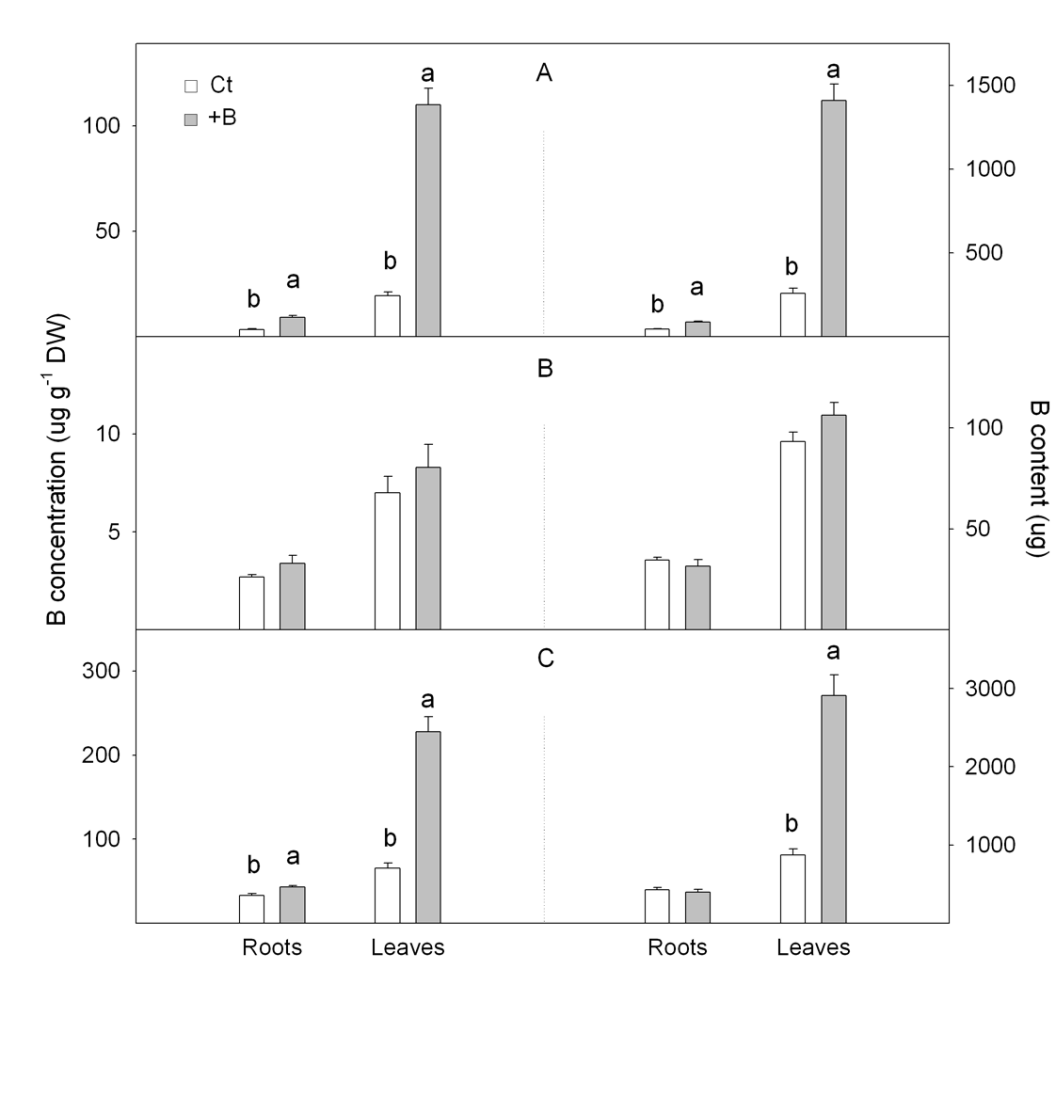
Boron concentration ([B_f_], μg g^-1^ DW) and boron content (B_f_, μg) in (A) soluble in water, (B) soluble in organic solvents and (C) insoluble fractions measured in roots and leaves of *Citrus macrophylla* seedlings grown for 25 days in B-normal (50 μM, Ct) and B-toxic (400 μM, +B) nutrient solutions. Values are the means ± SE of three independent experiments (n = 3). For a comparison of means, an ANOVA followed by the LSD test, calculated at the 95% confidence level, was performed. Different letters indicate significant differences for each parameter and within each plant organ (P <0.05).
